# De Novo Transcriptome Assembly and Annotation of the Leaves and Callus of *Cyclocarya Paliurus* (Bata1) Iljinskaja

**DOI:** 10.1371/journal.pone.0160279

**Published:** 2016-08-02

**Authors:** Xiaoxiang Xu, Zhongping Yin, Jiguang Chen, Xiaoqiang Wang, Dayong Peng, Xinchen Shangguan

**Affiliations:** 1 Jiangxi Key Laboratory of Natural Products and Functional Food; Jiangxi Agricultural University, Nanchang 330045, China; 2 College of Food Science and engineering, Jiangxi Agricultural University, Nanchang 330045, China; 3 State Key Laboratory of Medicinal Chemical Biology and College of Pharmacy, Nankai University, Tianjin 300000, China; Youngstown State University, UNITED STATES

## Abstract

*Cyclocarya Paliurus* (Bata1) Iljinskaja contains various bioactive secondary metabolites especially in leaves, such as triterpenes, flavonoids, polysaccharides and alkaloids, and its leaves are widely used as an hyperglycemic tea in China. In the present paper, we sequenced the transcriptome of the leaves and callus of *Cyclocarya Paliurus* using Illumina Hiseq 4000 platform. After sequencing and de novo assembly, a total of 65,654 unigenes were generated with an N50 length of 1,244bp. Among them, 35,041 (53.37%) unigenes were annotated in NCBI Non-Redundant database, 19,453 (29.63%) unigenes were classified into Gene Ontology (GO) database, and 7,259 (11.06%) unigenes were assigned to Clusters of Orthologous Group (COG) categories. Furthermore, 11,697 (17.81%) unigenes were mapped onto 335 pathways in Kyoto Encyclopedia of Genes and Genomes (KEGG), among which 1,312 unigenes were identified to be involved in biosynthesis of secondary metabolites. In addition, a total of 11,247 putative simple sequence repeats (SSRs) were detected. This transcriptome dataset provides a comprehensive sequence resource for gene expression profiling, genetic diversity, evolution and further molecular genetics research on *Cyclocarya Paliurus*.

## Introduction

*Cyclocarya Paliurus* (Bata1) Iljinskaja, a unique genus of Juglangdaceae, is a well-known edible and medicinal plant growing in southern China. The leaves of *Cyclocarya Paliurus* have been often used to produce teas with health benefits, which is named “sweat tea” for its slight sweetness [1–4]. It has been demonstrated that *C*. *Paliurus* exhibits various pharmacological activities such as anti-hypertensive, hypoglycemic, antioxidant and enhancement of mental efficiency [5–7], which may be mostly attributed to its various bioactive components, *e*.*g*. flavonoids [4], triterpenoids [8], polysaccharides [9] and polyphenols [10]. Among these compounds, triterpenoids are an important group of health-promoting chemicals. In our previous studies, eight triterpenoids named β-amyrin, ursolic acid, oleanolic acid, betulinic acid, corosolic acid, maslinic acid, β-boswellic acid, and arjunolic acid were isolated and identified from the *C*. *Paliurus* leaves [11], which have been proven to have valuable pharmacological and biological activities [12–14], and are now widely used in drugs, food and cosmetics.

However, because of the great difficulty in propagation and cultivation, *C*. *Paliurus* is endangered, and there are very few populations scattered in remote forested mountains, which seriously impaired the utilization of this resource [14]. We investigated the cutting of *C*. *Paliurus* [15], and achieved a rooting rate of 64.57% by the stimulation of SJCL (a rooting agent) [16]. We studied another rapid propagation method named *in vitro* culture of stem segment and obtained a high bud ratio, but found it was hard to root and grow into a tree [17]. Although significant efforts have been made, applicable large-scale breeding and cultivation techniques of *C*. *Paliurus* have not been established yet. In order to alleviate the resource shortage, we tried to produce the *C*. *Paliurus* secondary metabolites by the plant cell culture. Our group succeeded in inducing callus and establishing the cell culture technology, and found cell culture was a promising way to yield triterpenic acids [18, 19]. Five triterpenic acids have been isolated and identified in the cell cultures of *C*. *Paliurus* in our recent studies [20].

However, there are few studies on the synthetic mechanism of *C*. *Paliurus* secondary metabolites, and very limited information is available about the metabolism and related biosynthetic genes. So far, only one *C*. *Paliurus* gene named CpFPS was reported [21]. It’s very hard to do more further researches at the molecular level on metabolic mechanism and regulation to achieve high metabolite production. RNA-seq (High-throughput RNA sequencing technology) provides us a feasible way to carry out some secondary metabolism investigations without genomic sequence, and therefore is particularly attractive for non-model organisms like *C*. *Paliurus*.

RNA-seq is a powerful tool for transcriptome analysis based on second-generation sequencing technology, which have already made substantial contributions to our understanding of genome expression and regulation [22]. This technology can be used to estimate the expression of genes or isoforms, detect differentially expressed genes, and determine novel splice junctions [22–24]. In recent years, RNA-seq has been widely applied in the genome-wide quantification of absolute transcript levels, and the mining of molecular markers and identification of genes involved in biosynthesis of various secondary metabolites in plants, such as *Salvia miltiorrhiza [25]*, *Panax notoginseng* [26], *Asparagus racemosus* [27], *Cunninghamia lanceolata* [28], *Gentiana rigescens* [29], and *Astragalus membranaceus* [30].

In the present study, high-quality transcriptome data of the leaves and callus from *C*. *Paliurus* were obtained using Illumina Hiseq 4000 platform, and a total of 65,654 assembled unigenes were generated and annotated against public protein databases followed by GO, COG and KEGG classification. Moreover, 11,247 putative simple sequence repeats (SSRs) were detected. These transcriptome data provide a valuable public genomic resource for understanding the metabolic mechanisms and facilitating the discovery of genes involved in secondary metabolism pathway and its regulatory, as well as the future gene expression profiling, functional genomic studies of *C*. *Paliurus*.

## Materials and Methods

### Plant Material and RNA Extraction

The *C*. *Paliurus* leaves for RNA-seq were harvested from the arboretum of Jiangxi Agricultural University in July. Calluses were induced from the leaves collected between April and May, then inoculated on fresh agar-based MS medium (Murashige and Skoog medium) [31] supplemented with 2,4-dichlorophenoxy (2,4-D 0.5 mg/L), 1-Naphthaleneacetic acid (NAA 0.3 mg/L) and 6-Furfurylamino-purine (KT 1.0 mg/L) and cultured under a 12/12 h (light/dark) photoperiod. The subculture interval was initially 20 days, then gradually decreased to 10 days with the increase of subculture time [32]. The collected samples were immediately frozen in liquid nitrogen and stored at -80°C. Total RNA of each sample were extracted and purified using TRIzol^®^ reagent (Plant RNA purification reagent, Invitrogen, Carlsbad, CA, USA) according the manufacturer’s instructions. The RNA concentration and purity were detected by Nanodrop 2000 (Thermo Fisher, America), and the quality of RNA was further verified by gel electrophoresis and Agilent 2100. Only high-quality RNA samples (OD 260/280 = 1.8~2.2, OD260/230≥2.0, RIN≥6.5, 28S:18S≥1.0, >10μg) were used to construct sequencing library.

### cDNA library construction and Illumina sequencing

RNA-seq transcriptome library was prepared from 5μg of total RNA using TruSeq^TM^ RNA sample preparation kit from Illumina (San Diego, CA). The poly(A) mRNA was isolated from total RNA using Oligo (dT) magnetic beads. Following purification, the mRNA was randomly cleaved into short fragments (100 to 400 bp) after adding fragmentation buffer. These short fragments were used as templates to synthesize the first-strand cDNA using reverse transcriptase and random primers. The second-strand was synthesized subsequently using a SuperScript double-stranded cDNA synthesis kit (Invitrogen, CA) with random hexamer primers (Illumina). The cDNA fragments were purified and resolved with EB buffer for end repair and A-tailing addition, and then connected with paired-end adapters. After PCR amplification using Phusion DNA polymerase (NEB) for 15 PCR cycles, the cDNA target fragments of 200–300 bp were size-selected to establish the cDNA library on 2% Low Range Ultra Agarose. After quantification by TBS380, the paired-end RNA-seq sequencing library was sequenced from the 5' to 3' ends using an Illumina Hiseq 4000 platform with the 2×151 bp paired-end read module.

### Data filtering and De novo assembly

The raw paired-end reads, which were transformed by the Base Calling into sequence data, were cleaned to high-quality reads by removing the joint sequences and adaptor sequences, reads containing more than 10% N rate, and trimming the low-quality reads (quality value < 20) from the 3' end of the sequence and the raw reads with an average length less than 30bp. Then, de novo transcriptome assembly was conducted using software Trinity (http://trinityrnaseq.sourceforge.net/, Version number: trinityrnaseq, release-20140413) without reference genome [33], Trinity consists of three software modules: Inchworm, Chrysalis and Butterfly, and has been regarded as the authoritative software for the efficient and robust de novo reconstruction of transcriptome.

De novo assembly was carried out according to the established method [33], which was briefly described as follows. Firstly, linear transcript contigs were efficiently reconstructed by Inchworm in the following seven steps: (1) Constructing a k-mer dictionary (k = 25) from all sequence reads; (2) Removing likely error-containing k-mers from the dictionary; (3) Selecting the most frequent k-mer to seed a contig assembly (excluding both low-complexity and singleton k-mers); (4) Extending the seed in each direction with the highest occurring k-mer of a k–1 overlap; (5) Extending the sequence in either direction until it cannot be extended further; (6) reporting the linear contig; (7) Repeating steps three to six with the next most abundant k-mer until the entire k-mer dictionary has been exhausted. Secondly, Inchworm contigs were recursively grouped into connected components by Chrysalis. Contigs with a perfect overlap of k– 1 bases or a minimal number of reads that span the junction across both contigs were deemed to be derived from the same gene and clustered into the same group. After grouping, complete de Bruijn graphs were constructed for each component. Thirdly, Butterfly processed the individual graphs independently, and extracted full-length isoforms and teased apart transcripts derived from paralogous genes. Redundant sequences were eliminated, and the longest transcript that could not be extended on either end was defined as unigenes. The assembled unigenes (longer than 200 bp) had been deposited into the NCBI Transcriptome Shotgun Assembly Sequence Database (http://www.ncbi.nlm.nih.gov/genbank/tsa/) with the accession numbers (GEUI00000000).

### Functional annotation and classification

All assembled unigenes were aligned with BLASTX program for homology searches against publicly available protein databases Non-redundant (http://www.ncbi.nlm.nih.gov/), Swissprot (http://www.ebi.ac.uk/uniprot/), Pfam (http://pfam.sanger.ac.uk/), String(http://string-db.org/) with identity set at >30% and a cutoff E-value of 10^−5^, and annotated and classified on Gene Ontology (http://www.geneontology.org/), Clusters of Orthologous Group (http://www.ncbi.nlm.nih.gov/COG/), and the KEGG pathway (http://www.genome.jp/kegg/) with a threshold E-value of 10^−5^. The aligning results were used to identify the sequence direction and to predict the coding regions. If the aligning results from different databases were conflicted with each other, a priority order of alignments from Nr, SwissPort, KEGG, GO and COG was followed. Based on the Nr annotations, the Blast2GO program was used to obtain GO annotations according to biological process, molecular function and cellular component [34]. The unigenes were also aligned to the COG database to predict and classify functions, and the secondary metabolic pathways were annotated according to the KEGG pathway database. Transcription Factors of *C*. *Paliurus* were extracted from Plant Transcription Factor Database (PlantTFDB), and unigenes were mapped to them using Blastn program.

### Detection of SSR markers

The unigenes were scanned for microsatellites using the MISA software (http://pgrc.ipkgatersleben.de/misa/) with the default parameters. The parameters were adjusted for identification of perfect di-nucleotide, tri-nucleotide, tetra-nucleotide, penta-nucleotide, and hexa-nucleotide motifs with a minimum of 6, 5, 5, 5, and 5 repeats, respectively. Primer pairs were designed using Primer 3.0.

## Results and Discussion

### RNA sequencing and de novo assembly

**Fig 1:**
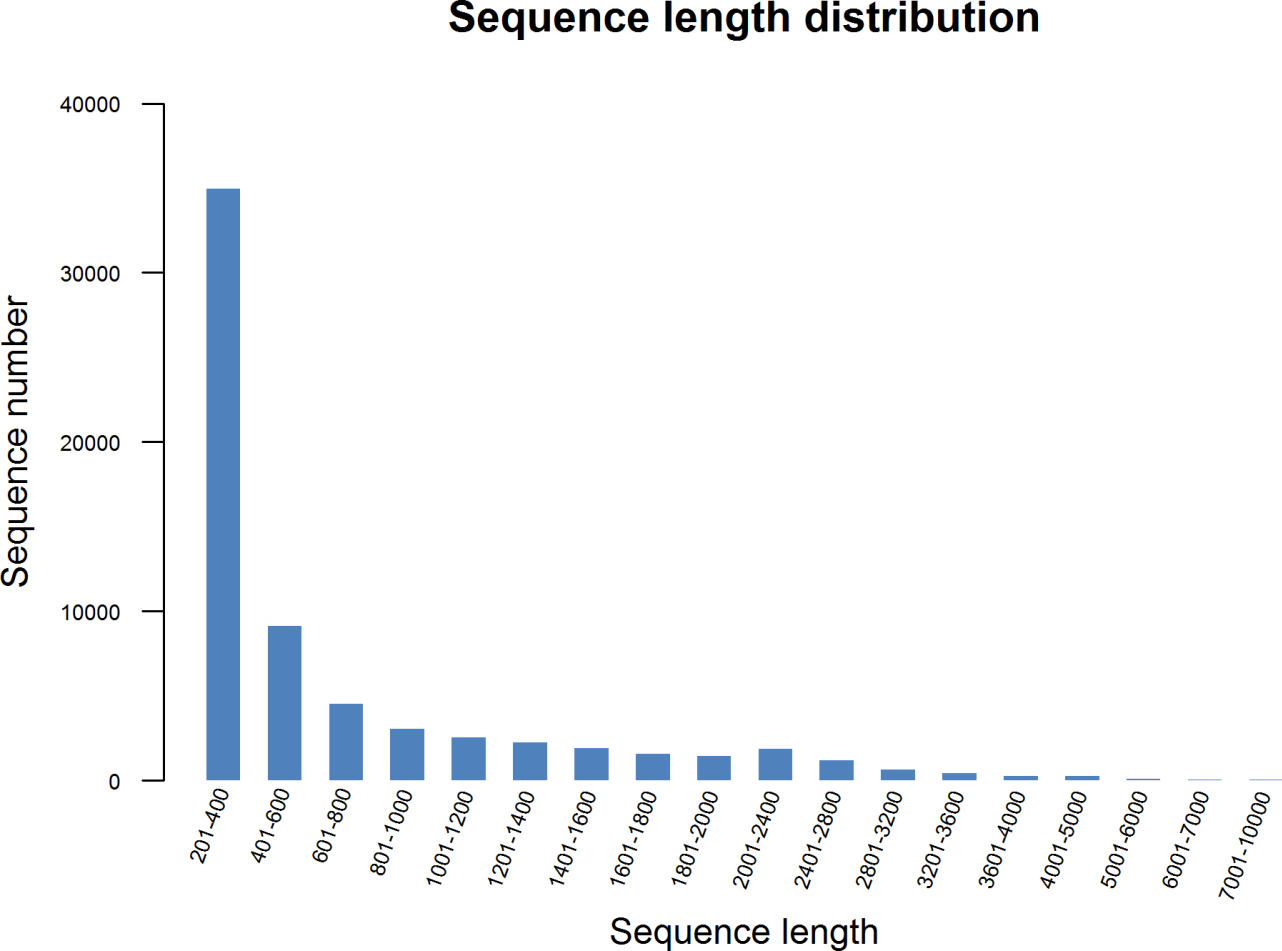
Length distribution of assembled unigenes. Among 65,654 unigenes, 44,010 (67.03%) were shorter than 600bp and 14,155(21.56%) were longer than 1,000bp.

To generate a comprehensive overview of *C*. *Paliurus* transcriptome, total RNA were extracted from leaves and callus, then the mRNA was isolated, and cDNA libraries were established and sequenced separately using Illumina Hiseq 4000 platform, which generated 39.0 and 49.4 million raw reads, respectively (Table 1). After removing adaptor sequences, ambiguous reads and low-quality reads, the quality of reads was assessed successively. The clean reads were individually generated with the average GC percentage of 46.49% and 47.90% (Table 1). By using the Trinity program [1], all high-quality reads were assembled into 65,654 unigenes with an N50 of 1,244 bp and average length of 704 bp, and 84,223 transcripts were constructed with an N50 of 1,362 bp and average length of 792 bp (Table 2). The average GC content of the unigenes was 42.95%. Furthermore, the length of these unigenes ranged from 201 to 10,000 bp (Fig 1), and the majority were disturbed in 201-400bp. However, there are still 14,155 unigenes (21.56%) whose lengths were more than 1,000bp. These data indicated that the generated unigenes in our experiments were of fine quality and therefore suitable for further annotation.

**Table 1:** Summary of transcriptome sequencing of *C*. *Paliurus* leaves and callus.

	leaves	callus
Total raw reads	39,053,944	49,408,746
Total clean reads	38,324,176	48,392,438
Q20 percentage (%)	98.11	98.09
Q30 percentage (%)	94.18	94.12
Error percentage (%)	0.0116	0.0117
GC percentage (%)	46.49	47.90

(Note: Q20, the proportion of nucleotides with quality value larger than 20 in reads, Q30, the proportion of nucleotides with quality value larger than 30 in reads)

**Table 2:** Summary of the sequence assembly after Illumina sequencing.

	Unigenes	Transcripts
Total sequence num	65,654	84,223
Total sequence base	46,199,033	66,728,179
GC percentage (%)	42.94	42.96
Average length (bp)	704	792
Smallest length (bp)	201	201
Largest length (bp)	9184	9184
N50 (bp)	1,244	1,362

### Functional annotation and classification

**Fig 2:**
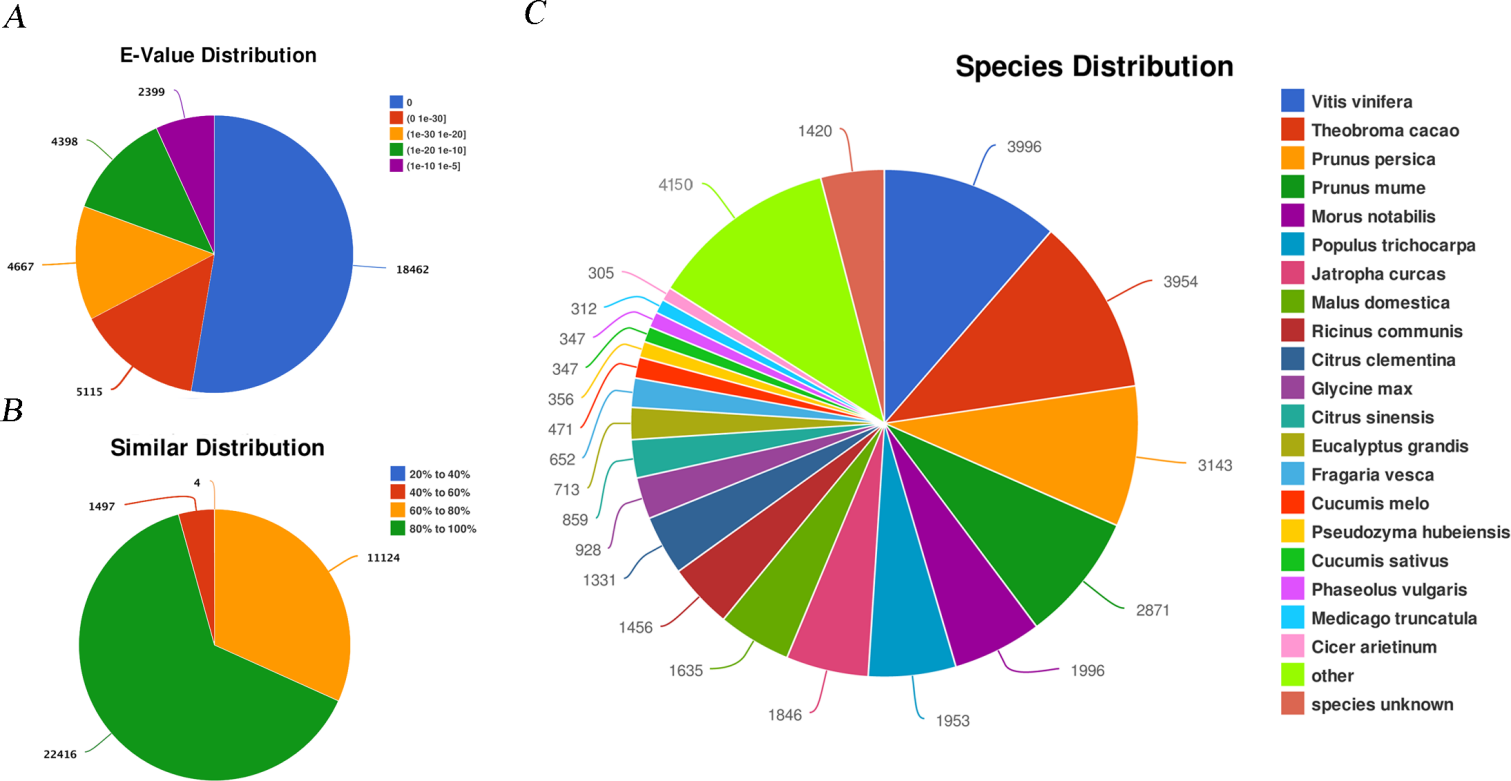
Unigenes homology searches against the NR database. (A) The E-value distribution of BLAST hits. (B) The similar distribution of BLAST hits. (C) Species distribution of the top BLASTX hits.

In order to predict and analyze the function of assembled unigenes, the total annotated unigenes were aligned against the NCBI non-redundant (Nr) database, the String and SwissPort protein database, the Gene Ontology (GO) database, the Clusters of Orthologous Group (COG) and Kyoto Encyclopedia of Genes and Genomes (KEGG) database using the BLASTX program with an E-value cut-off of 10^−5^. In total, 35,041 (53.37%) unigenes were annotated in Nr database (S1 Table). Beyond that, 17,709 (26.97%), 15,529 (23.65%), 11,697 (17.81%), 20,629 (31.42%) unigenes were annotated in Pfam, String, KEGG, SwissPort databases, respectively (Table 3). Further blast statistics indicated that 80.60% of the annotated unigenes in NR exhibited high homology with the E-value < 1e-20, and 52.69% with a strong E-value (E-value = 0) (Fig 2A). The similarity distribution showed that 63.97% of the annotated sequences had similarities higher than 80%, while 36.02% had a similarity between 40% and 80% (Fig 2B). Additionally, the annotated unigenes were compared to known nucleotide sequences of other plant species, which were best matched to the known nucleotide sequences from Vitis vinifera (11.40%), followed by Theobroma cacao (11.28%), Prunus persica (8.97%), Prunus mume (8.19%), and Morus notabilis (5.69%) (Fig 2C).

**Table 3:** Summary of unigenes annotation.

Database	Total unigenes	Annotated unigenes	Percentage
Pfam	65,654	17,709	26.97%
String	65,654	15,529	23.65%
KEGG	65,654	11,697	17.81%
Swissprot	65,654	20,629	31.42%
NR	65,654	35,041	53.37%

**Fig 3:**
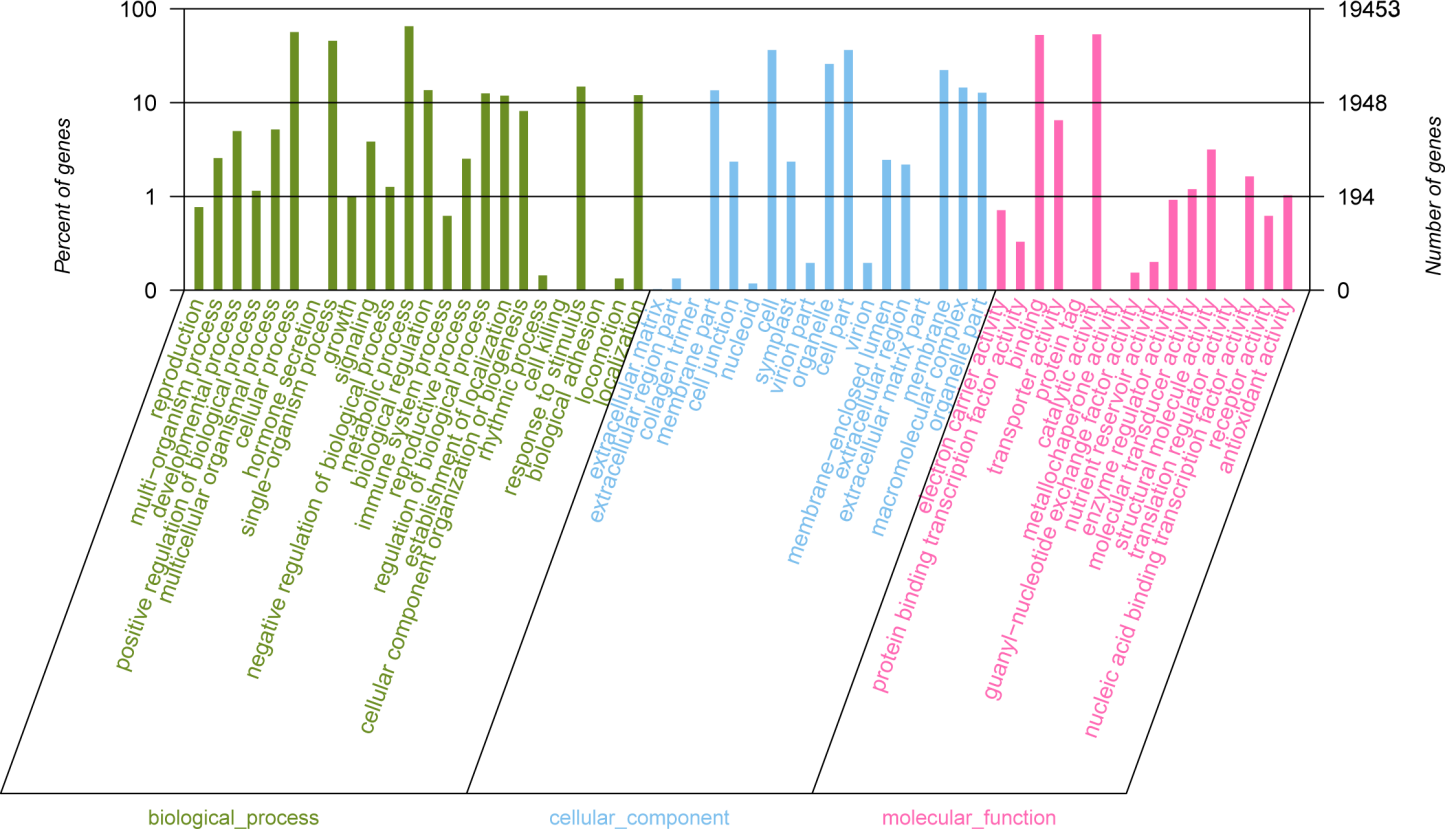
GO classification of assembled sequences. A total of 19,453 unigenes were classified into three main GO categories: Biological Processes, Cellular Component, and Molecular Function.

Gene Ontology (GO) is an international standardized gene functional classification system. The GO terms for *C*. *Paliurus* unigenes were retrieved using Blast2GO [35]. A total of 19,453 (29.63%) assembled unigenes were annotated and classified into three main categories: Biological Processes, Cellular Component, and Molecular Function, and then distributed into 58 sub-categories (Fig 3). Within the Biological Processes classification, metabolic process (12,696, 65.32%), cellular process (11,001, 56.59%), and single-organism process (8,854, 45.60%) were the most significantly represented (Fig 3), which indicated that these unigenes played an important metabolic activity in *Cyclocarya Paliurus*. Under the Cellular Component classification, the unigenes were mainly related to “cell” (7,090, 36.45%) and “cell part” (7,090, 36.45%), followed by “organelle” (5,031, 25.86%) and “membrane” (4,324, 22.23%). Only a few unigenes were assigned to extracellular matrix, extracellular matrix part and collagen trimer. In the Molecular Function category, the majority were assigned to “binding” (10,265, 52.77%) and “catalytic activity” (10,428, 53.61%) prominently. The above-mentioned findings were similar to the recent report of Chinese Chive transcriptome functional annotation [35].

**Fig 4:**
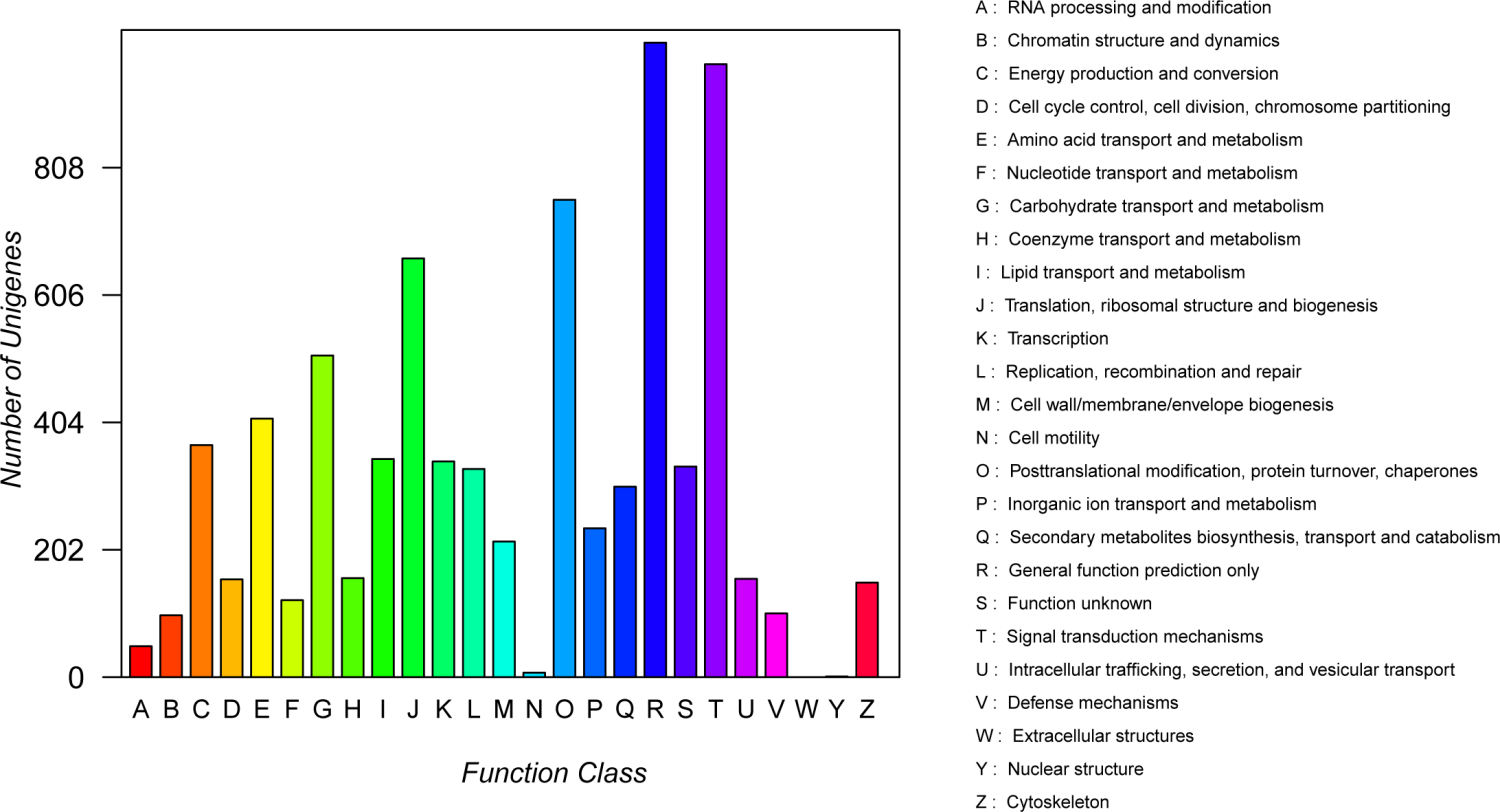
COG functional classification. A total of 7,259 unigenes were assigned to 25 classification categories.

COG is a database which is widely used to predict and classify functional genes. Every protein in the COG database is assumed to be evolved from an ancestor, and the whole database is built on coding proteins with complete genomes as well as system evolution relationships of bacteria, algae, and eukaryotes [36, 37]. Out of the 65,654 unigenes, 7,259 (11.06%) were annotated and classified into 24 functional categories (Fig 4). Among the aligned COG classifications, “general function prediction only” category (1,006, 13.86%) was the largest group, followed by “signal transduction mechanisms" (972, 13.39%), “posttranslational modification, protein turnover, chaperones” (757, 10.43%), “translation, ribosomal structure and biogenesis” (664, 9.15%), and “carbohydrate transport and metabolism” (510, 7.03%), whereas only few unigenes were assigned to “cell motility” (7, 0.096%), “extracellular structures” (1, 0.014%), and “nuclear structure” (1, 0.014%), respectively. In addition, 334 unigenes were classified into the unknown function.

### Metabolic pathway analysis by KEGG

**Fig 5:**
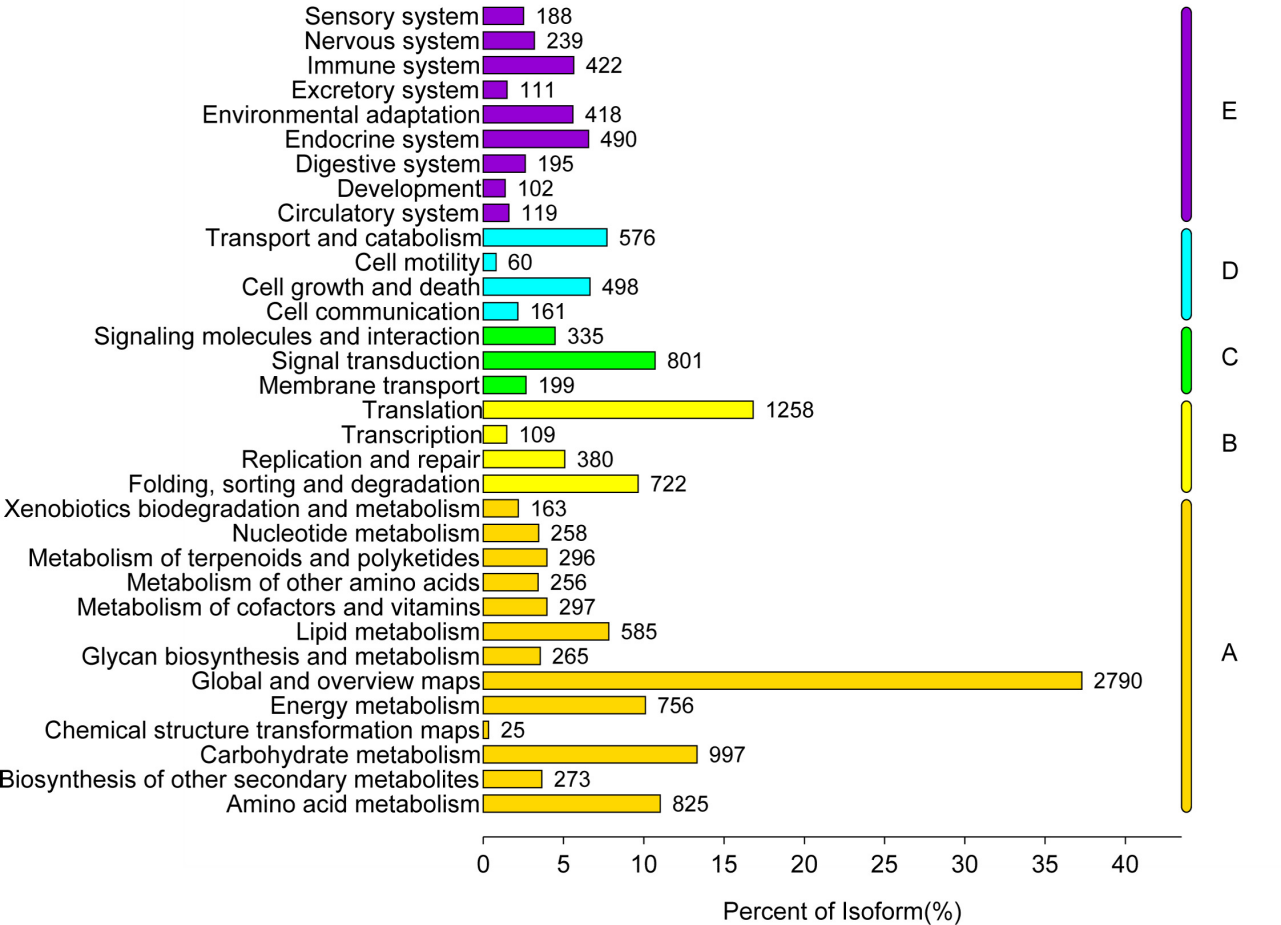
KEGG pathway annotation of the unigenes. These unigenes were divided into five branches (A, Metabolism; B, Genetic Information Processing; C, Environmental Information Processing; D, Cellular Processes; E, Organismal System.)

**Fig 6:**
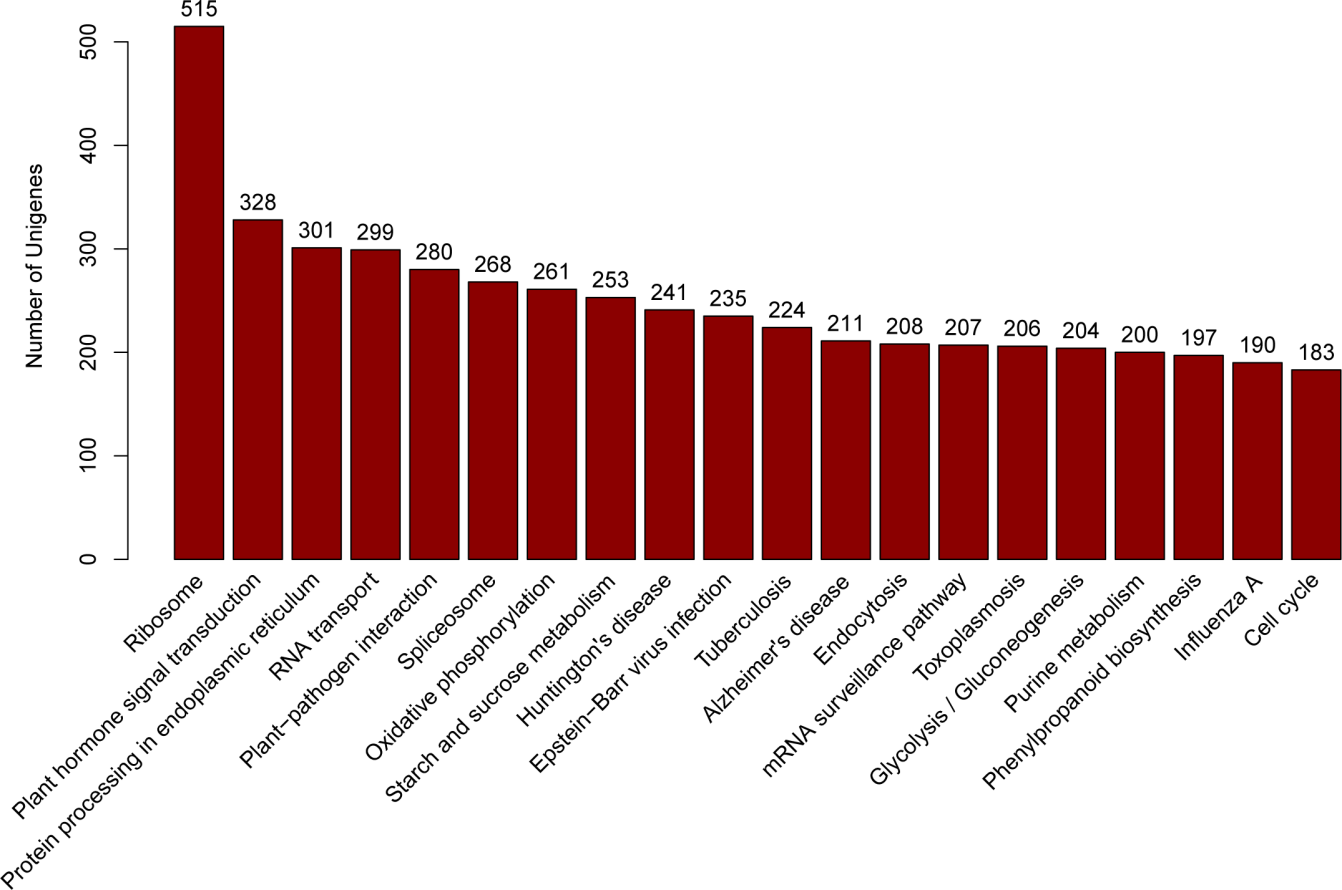
The top 20 largest KEGG pathways. Annotated unigenes were classified into 335 KEGG pathways. The top 20 pathways containing unigenes are displayed.

The KEGG pathway database provides a wealth of information on molecular interaction and reaction networks t o further understand the biological functions of unigenes. The mapped results indicated that 11,697 (17.81%) unigenes were predominantly annotated with Enzyme Commission (EC) numbers and divided into five branches according to the metabolic pathway (Fig 5) (Metabolism; Genetic Information Processing; Environmental Information Processing; Cellular Processes; Organismal Systems), and further grouped into 335 KEGG pathways (S2 Table). It was noteworthy that 7,746 (66.34%) mapped unigenes participated in the metabolism and 1,312 (11.24%) were involved in the biosynthesis of secondary metabolites such as phenylpropanoid biosynthesis (197), flavonoid biosynthesis (42), terpenoid backbone biosynthesis (58), which were the important information we are especially interested in. Furthermore, the top 20 largest annotated pathway groups of *C*. *Paliurus* were presented in Fig 6. The most representative KEGG pathway was “Ribosome”, followed by “Plant hormone signal transduction” (328), “Protein processing in endoplasmic reticulum” (301), “RNA transport” (299), and “Plant pathogen interaction” (280). As shown in Fig 6, there were 328 (2.81%) unigenes mapped into the “Plant hormone signal transduction” pathway, some of which might be stimulated to express by the supplemented plant hormones in the callus culture medium. In our experiments, plant hormones such as rootone, indole acetic acid, and cytokinins were added into the callus culture medium, which have been presumed to participate in the regulation of “Plant growth”, “Cell culture”, “Cell differentiation”, and “Recession”. These predicted results indicated that numerous unigenes were involved in the secondary metabolite biosynthesis of *C*. *Paliurus* leaves and callus. The above mapped information with KEGG pathway database would be very useful for future researches on gene function and its regulatory mechanism of *C*. *Paliurus*.

### Transcription factor analysis

**Fig 7:**
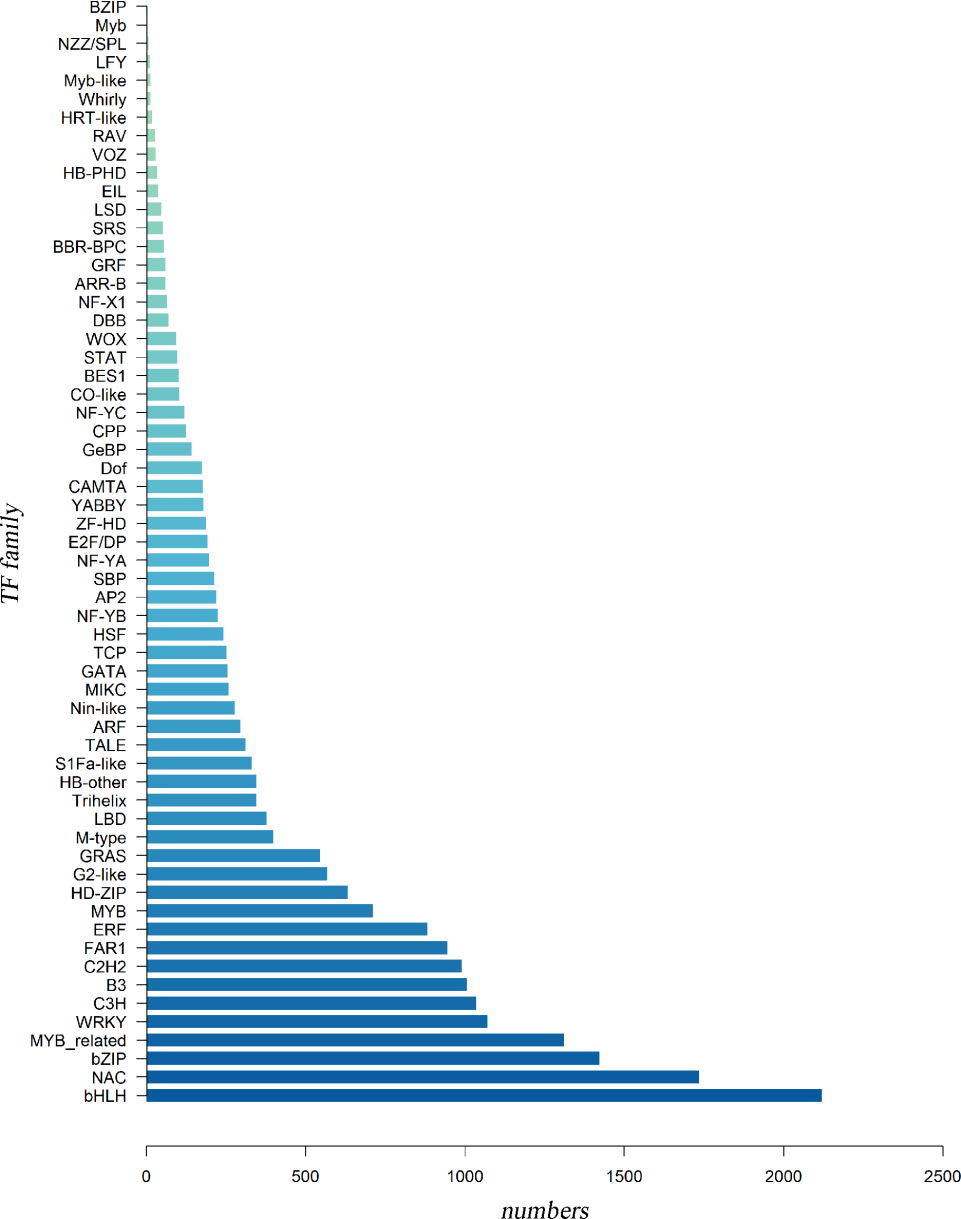
Summary of transcription factor unigenes of *C*. *Paliurus*. The number of unigenes related to TFs in each TF family. Among the TF families bHLH, NAC, bZIP and MYB_related proteins were the most abundant.

Transcription factors (TFs) play critical roles in plant growth, bioactive component synthesis and gene expression regulation, especially in the secondary metabolism regulation. Plants show various TFs expression patterns when growing in different environments or facing stress, which further significantly affect the synthesis of secondary metabolites [38–40]. Therefore, the identification of putative TF genes is useful for understanding the regulatory mechanism of secondary metabolites. TFs are often classified into different families according to the features of DNA-binding domains. In this study, a total of 21,843 unigenes were annotated and further classified into 60 transcription factor families (Plant Transcription Factor Database, PlantTFDB, http://planttfdb.cbi.pku.edu.cn) in this paper (Fig 7). Among these TF families, the most abundant transcription factors of *C*. *Paliurus* were found in the bHLH family which includes 2,120 unigenes. The second was NAC, followed by the bZIP, MYB-related, WRKY, C3H, B3 and C2H2 TF families, which contains 1,734, 1,422, 1,311, 1,071, 1,036, 1,007 and 990 unigenes, respectively. Researches have validated that the bHLH, WRKY, MYB, bZIP, and C2H2 TF families play a major role in the regulation of many genes which participate in the plant secondary metabolism [41, 42], especially for the regulation of the bioactive component synthesis, such as flavonoids [43], alkaloids [44, 45], and terpenoids [46]. The expression level of these TFs in *C*. *Paliurus* may be associated with the biosynthesis of secondary metabolites, and the discovery of these putative TFs may provide valuable information for the future researches on gene expression regulation, particularly those TFs related to the flavonoid pathway which will be described below.

### Analysis of flavonoid biosynthesis pathway

**Fig 8:**
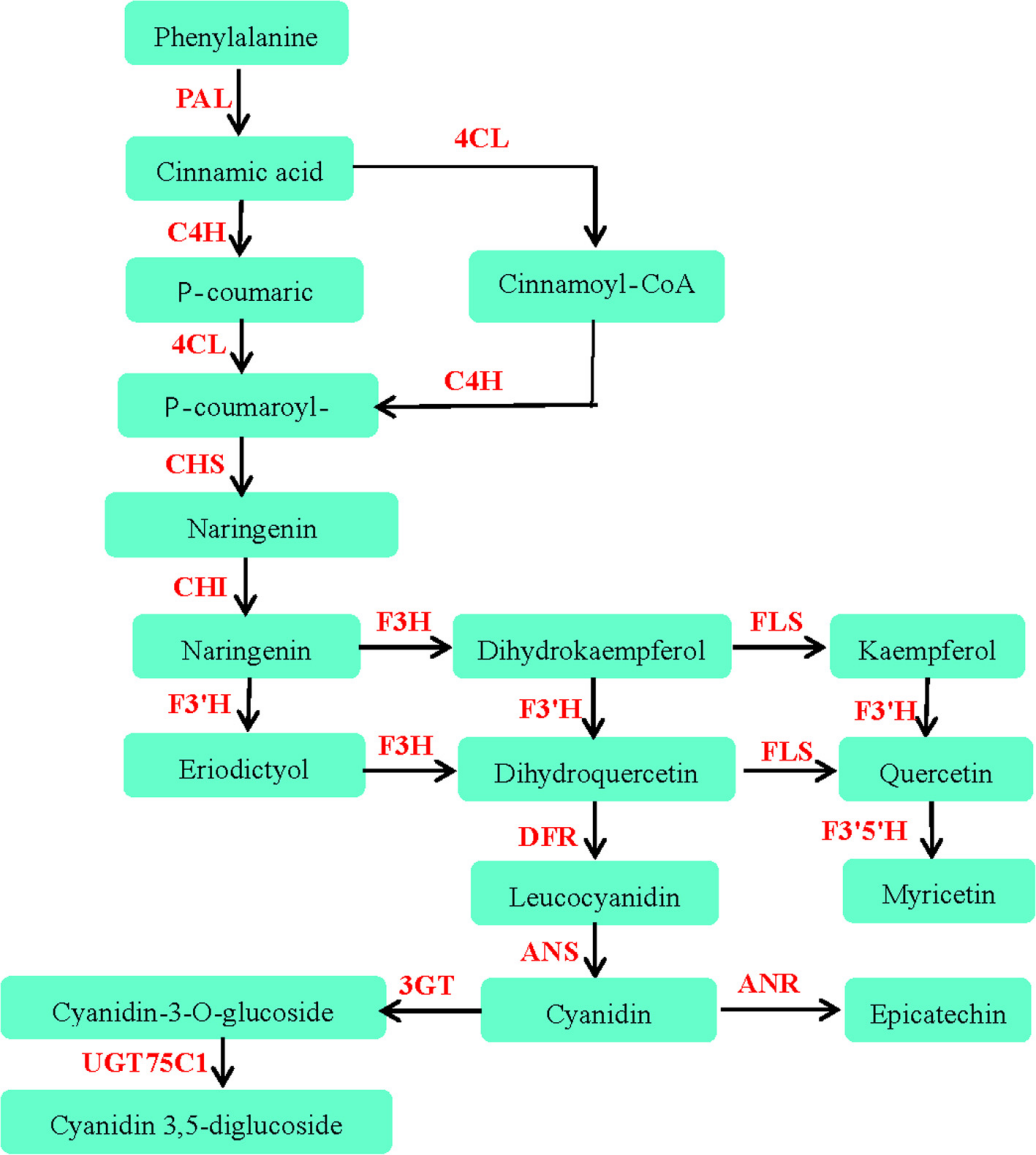
Putative biosynthesis pathway of flavonoids and phenolic compounds in *C*. *Paliurus*. PAL, phenylalanine ammonialyase; C4H, cinnamate-4-hydroxylase; 4CL,4- coumaroyl:coenzyme A ligase; CHS, chalcone synthase; CHI, chalcone isomerase; F3H, flavanone -3- hydroxylase; DFR, dihydroflavonol 4-reductase; FLS, flavonol synthase; ANS, anthocyanidin synthase; LAR, leucoanthocyanidin reductase; ANR, anthocyanidin reductase; 3GT, anthocyanidin 3-O-glucosyltransferase; F3'5'H, flavonoid 3',5'-hydroxylase and UGT75C1,UDP-glucose: anthocyanidin 3-O-glucoside 5-O-glucosyltransferase.

Flavonoids are polyphenolic secondary metabolites derived from the phenylalanine via the phenylpropanoid pathway, which become a research focus in recent years for their various biological and pharmacological activities [47]. It was reported that the total flavonoid content of *C*. *Paliurus* leaves ranged from 0.73 to 4.73%, depending on the growing region, harvest time, determination method and so on [48–50]. The most abundant flavonoid was isoquercitrin in the *C*. *Paliurus* leaves [12]. Up to now, sixteen flavonoids have been isolated and identified from *C*. *Paliurus* (Table 4). In the present study, 197 unigenes were found to be involved in phenylpropanoid pathway by mapping with KEGG pathway database. Among them, 42, 3, 1 and 3 unigenes were involved in the biosynthesis of flavonoids, anthocyanin, isoflavonid and flavone and flavonol, respectively, which represented different enzymes in the different pathways. The unigenes associated with flavonoid biosynthetic pathway were shown in the Fig 8. A total of 10 candidate genes with annotations matching enzymes in the flavonoid biosynthesis, i.e., phenylalanine ammonialyase (PAL), cinnamate-4-hydroxylase (C4H), 4- coumaroyl:coenzyme A ligase(4CL), chalcone synthase (CHS), chalcone isomerase (CHI), flavanone -3- hydroxylase (F3H), flavanone-3'-hydroxylase (F3'H), flavonoid 3',5'-hydroxylase (F3'5'H), dihydroflavonol 4-reductase (DFR) and flavonol synthase (FLS), were annotated in this paper. Moreover, the unique putative unigenes encoding anthocyanidin synthase (ANS), leucoanthocyanidin reductase (LAR), anthocyanidin reductase (ANR) and anthocyanidin 3-O-glucosyltransferase (3GT) were also prominently found. It's important to clone these unique genes and further analyze their functions in the future studies. Isoflavonoids, a subclass of flavonoids with special structure and function, have been normally found in leguminous plants. It's interesting that one isoflavonoid 7-hydroxyl-4´-methoxy isoflavone was identified in *C*. *Paliurus* leaves [51]. However, 2-hydroxyisoflavanone synthase (IFS), which catalyzes the conversion of flavanones to isoflavones, hasn’t been found in our mapping experiments. A further study is needed in the future.

**Table 4:** Summary of flavonoids identified in *C*. *Paliurus*.

Number	Name	Chemical Formula	Reference
**1**	Kaempferol	C_15_H_10_O_6_	[71]
**2**	Quercetin	C_15_H_10_O_7_	[71]
**3**	Isoquercitrin	C_21_H_20_O_12_	[71]
**4**	3,6,3',5'-tetramethoxy-5,7,4' trihydroxyflavone	C_19_H_18_O_9_	[72]
**5**	Kaempferol-7-O-α-L-rhamnoside	C_21_H_20_O_10_	[73]
**6**	Kaempferol-4´-Methylether-7-O-β-D-Mannose	C_22_H_22_O_11_	[73]
**7**	Quercetin-3-O-α-L-rhamnopyranoside	C_21_H_20_O_11_	[74]
**8**	Kaempferol-3-β-D-glucuronide	C_21_H_18_O_12_	[75]
**9**	Kaempferol-3-O-α-L-Rhamnopyranoside	C_21_H_20_O_10_	[76]
**10**	Kaempferol-3-O-β-D-Galactopyranoside	C_21_H_18_O_11_	[76]
**11**	Kaempferol 3-O-β-D-glucopyranoside	C_21_H_20_O_11_	[76]
**12**	Kaempferol-3-O-α-L-(4′′-E-p-coumaroyl) rhamnopyranoside	C_33_H_32_O_15_	[76]
**13**	Quercetin-3-O-β-D-glucuronate sodium	C_21_H_17_O_14_Na	[77]
**14**	myricetin-3-O-β-D-glucuronate sodium	C_15_H_10_O_15_Na	[77]
**15**	7-hydroxyl-4´-methoxy isoflavone	C_21_H_17_O_15_	[51]
**16**	Myricetin	C_15_H_10_O_8_	[51]

Transcription factors play an important role in flavonoid biosynthesis. These factors regulate some genes co-expression to stimulate or inhibit the accumulation of flavonoids when combining with the special structure gene function. So far, researchers have isolated and identified many transcription factor genes involved in regulation of flavonoids in *Maize* [52], *Arabidopsis* [53], *Petunia* [54], *Rice* [55] and other crops, such as MYB, MYC, bZIP, WD40, zinc finger TFs. Among these transcription factors, MYB and MYC (bHLH) families became the research focus.

MYC (bHLH) is one of the biggest transcription factor families in plant. This family regulates not only the plant growth and developmental processes including formation of trichome and light signal transduction, but also the stress responses and secondary metabolism [56, 57]. Plant bHLH proteins have been classified into 32 subfamilies according to genome-wild classification and the evolutionary analysis [58], whereas members of the same plant bHLH subfamilies had similar functional characteristic [59]. There were some bHLH subfamilies involved in the regulation of phenylpropanoid and terpenoids biosynthesis pathways, such as AtTT8 in *Arabidopsis* [60], OsRa-c in rice [61] and TcJAMYC in yew [46]. Furthermore, the above mentioned subfamilies have also been found to be related to the regulation of anthocyanin biosynthesis in various plants, for example, *Zea mays L*, *Oryza sativa L*, *Dahlia variabilis Hort*.and *Brassica oleracea L* [62–65].

It was reported that there were approximately 197 and 155 MYB genes in Arabidopsis and rice, respectively [66]. The first plant MYB gene, C1, encodes a MYB-like TF, was isolated from *Zea mays*, which regulated the synthesis of anthocyanin with the synergy of R/B proten family [67]. Grotewold et al characterized the maize P gene encode proteins with homology to the DNA-binding domain of MYB-like transcription factors [68]. According to Grotewold et al [69], maize P1 (R2R3-MYB transcription factor) improved the expression of CHS (chalcone synthase)、CHI (chalcone isomerase)、DFR(dihydroflavonol 4-reductase), but didn't stimulate the expression of branching enzyme such as F3H (flavanone 3-hydroxylase) in anthocyanin biosynthesis. However, it has been reported that many R2R3-MYB transcription factors, e.g., AN2 in Petunia hybrid [70] and PAP1/PAP2 in *Arabidopsis thaliana* [53], were involved in the regulation of anthocyanin biosynthesis. In our analysis, a total of 711 MYB genes were found in *C*. *Paliurus*. Therefore, more study remains to be done to identify the MYB genes from *C*. *Paliurus*for studying the regulation of flavonoid biosynthesis.

In the present paper, we found many transcription factors involved in flavonoid metabolism of *C*. *Paliurus*, providing critical information for further regulation study on the gene expression and secondary metabolism. We have established the cell suspension culture of *C*. *Paliurus* to produce the bioactive components such as flavonoids, triterpenoids and polysaccharides, and the above mentioned transcription factor information will be helpful to further develop and improve our current approach to achieve a higher yield of these compounds by gene expression regulation. Therefore, we can use genetic engineering for improving plant flavonoid secondary metabolic pathways to effectively increase the content of secondary metabolites by studying the transcription factors involved in flavonoid secondary metabolism, and understanding the mechanisms of plant secondary metabolic regulation.

### Detection of simple sequence repeats (SSRs)

SSRs, or microsatellites are important molecular markers for genetics and biology researches, including gene mapping, genetic diversity assessment, comparative genomics, and molecular breeding. In this paper, 65,654 assembled unigene sequences from *C*. *Paliurus* were scanned to explore the SSR profiles using MISA software and the results were shown in Table 5. A total of 9,688 sequences containing 11,247 SSRs were identified. Of all 9,688 SSR containing sequences, 1,353 had more than one SSR. In addition, 494 SSRs were present in compound forms. Among these SSRs, dinucleotide repeat motifs (5,198, 46.21%) were the most abundant, followed by 4,217 (37.49%) mononucleotide repeat motifs, 1,681 (14.95%) trinucleotide repeat motifs, tetra-nucleotide, hexa-nucleotide and penta-nucleotide repeat motifs. The main repeat motifs were AG/CT which accounted for 36.90% (4,150 SSRs), followed by A/T (4,004, 35.60%), AT/AT, AAG/CTT, AC/GT and ACC/GGT repeat (Table 6). A total of 5341 primer pairs, which contain three sets of primers respectively, were designed from 9,688 sequences using Primer 3 (S3 Table). These results provided plenty of reliable markers for genetic linkage mapping, analysis of genetic polymorphism and functional gene mining of *C*. *Paliurus* and its closely related species.

**Table 5:** Summary of the SSRs identified in the transcriptome sequences.

Item	Number
Total number of sequences examined	65,654
Total size of examined sequences (bp)	46,199,033
Total number of identified SSRs	11,247
Number of SSR containing sequences	9,688
Number of sequences containing more than 1 SSR:	1,353
Number of SSRs present in compound formation	494

**Table 6:** Frequency of SSRs repeat motifs in *C*. *Paliurus*.

Repeat motif length	Repeat number							Total	Frequency%
	5	6	7	8	9	10	>10		
AC/GT		134	73	45	22	12	21	307	2.72
AG/CT		1,109		737	746	879	679	4,150	36.90
AT/AT		216	132	121	113	110	29	721	6.41
CG/CG		10	8	2				20	0.18
AAC/GTT	44	18	5	1	1			69	0.61
AAG/CTT	283	170	100	5				558	4.96
AAT/ATT	77	25	21	1				124	1.10
ACC/GGT	96	35	23	3	1			158	1.40
ACG/CGT	26	10	7	4				47	0.42
ACT/AGT	17	9	5	1				32	0.28
AGC/CTG	126	40	25	1				192	1.71
AGG/CCT	146	65	46	4				261	2.32
ATC/ATG	107	48	30	4				189	1.68
CCG/CGG	35	6	10					51	0.45
Tetranucleotide		108	16		1			125	1.11
Pentanucleotide		11	1					12	0.11
Hexanucleotide			10	4				14	0.12

## Conclusions

*C*. *Paliurus* is a well-known edible and medicinal plant, but no genomic information is available yet. In this paper, the transcriptome of *C*. *Paliurus* leaves and callus without a reference genome was analyzed using Illumina Hiseq 4000 platform. A total of 65,654 assembled unigenes were generated. The annotated unigenes were functionally classified in the GO, COG, and KEGG databases. Moreover, the putative simple sequence repeats (SSRs) were detected. To our knowledge, this is the first attempt to de novo assemble the whole transcriptome of *C*. *Paliurus*. This study provided not only a comprehensive enough coverage for gene cloning, expression, and functional analysis, but also a valuable public platform to understand the biosynthesis and regulation of secondary metabolites in *C*. *Paliurus*, especially those important bioactive components.

## Supporting Information

S1 Table Unigene annotation by the NCBI NR, Swiss-Port, Pfam, String, COG, GO and KEGG databases. (XLSX)

S2 Table List of KEGG pathway in *C*. *Paliurus*. (XLSX)

S3 Table Designed SSR primers for *C*. *Paliurus*. (XLSX)
